# Subcutaneous emphysema as a complication of measles: A case series of nine pediatric patients from Ethiopia

**DOI:** 10.1016/j.idcr.2026.e02679

**Published:** 2026-07-10

**Authors:** Abel A. Gelan, Mikiyas G. Teferi, Nahom D. Gerer, Caleb M. Hailemariam, Leul M. Manyazewal, Mekbul Mohammed Aman

**Affiliations:** a Tulane University School of Public Health and Tropical Medicine, New Orleans, LA, USA; b School of Medicine, College of Health Sciences, Addis Ababa University, Addis Ababa, Ethiopia

**Keywords:** Measles, Subcutaneous emphysema, Pneumomediastinum, Malnutrition, Pediatrics, Ethiopia

## Abstract

Subcutaneous emphysema is an uncommon complication of measles with potentially serious consequences. This case series describes nine pediatric patients who developed subcutaneous emphysema during a measles outbreak at Bachuma Primary Hospital in rural Ethiopia between October and December 2023. Among 589 children admitted with complicated measles, nine developed subcutaneous emphysema, corresponding to an incidence of 1.52%, at the higher end of previously reported outbreak ranges. All patients were unvaccinated, with a median age of 24 months. Five children had acute malnutrition, including three with severe acute malnutrition and two with moderate acute malnutrition, and eight had clinical signs of micronutrient deficiency. Subcutaneous emphysema manifested a median of 4 days after rash onset, often after initiation of humidified nasal oxygen at 2–6 L/min. Management included supportive care, vitamin A, antibiotics when clinically indicated, nutritional rehabilitation, close monitoring, and emergency decompression when pneumothorax was suspected. Five patients were transferred to a regional hospital; four recovered and one died within 48 h after transfer from hypoxemic respiratory failure. This series highlights the importance of vigilance for air-leak complications in children with severe measles pneumonia, malnutrition, and oxygen requirement in resource-limited outbreak settings.

## Introduction

Measles remains a significant cause of childhood morbidity and mortality globally, particularly among unvaccinated children in resource-limited settings [1], [2]. Despite being vaccine-preventable, outbreaks continue to occur with substantial consequences in regions characterized by malnutrition, delayed healthcare access, and low immunization coverage. Measles complications include pneumonia, laryngotracheobronchitis, diarrhea, and encephalitis, which account for most measles-associated deaths [1], [3].

Subcutaneous emphysema is an uncommon but potentially serious complication defined by the presence of air in subcutaneous tissues, often manifesting as palpable crepitus on physical examination [3]. Historical reports indicate that subcutaneous emphysema complicates measles in approximately 0.59%−2.5% of cases during outbreaks, though incidence varies by setting and patient population [4], [5], [6], [7], [8]. The pathophysiology is commonly attributed to the Macklin phenomenon, whereby increased alveolar pressure from severe coughing, respiratory distress, or positive-pressure ventilation leads to alveolar rupture with subsequent air dissection along bronchovascular sheaths into the mediastinum and subcutaneous tissues [9], [10].

Several factors may predispose patients to this complication. Malnutrition impairs immune function and tissue repair, potentially increasing vulnerability to severe measles complications and air-leak phenomena [11], [12]. Severe pneumonia can generate forceful coughing and respiratory distress, elevating intra-alveolar pressures and creating conditions conducive to alveolar rupture [12], [13]. Young age may also contribute because children have more compliant chest walls and immature lung parenchyma [7].

The role of high-flow oxygen therapy warrants careful interpretation. Oxygen is essential for managing hypoxemia in severe measles pneumonia; however, higher flow rates can generate some degree of positive airway pressure and may contribute to barotrauma in vulnerable lungs with poor compliance [14], [15], [16]. In resource-limited settings where noninvasive ventilation, intubation, chest imaging, and critical-care transport resources may be unavailable, clinicians must balance oxygen support with close monitoring for air-leak complications.

Between October and December 2023, Bachuma Primary Hospital in the West Omo Region of Ethiopia experienced a measles outbreak affecting 589 hospitalized children. During this outbreak, nine children developed subcutaneous emphysema, corresponding to an incidence of 1.52%, at the higher end of the published outbreak range. We describe their clinical features, potential contributing factors, management, and outcomes.

## Case presentations

During the three-month outbreak period, 589 children were admitted with complicated measles to Bachuma Primary Hospital (Table 1). Among these children, nine developed subcutaneous emphysema, yielding an incidence of 1.52%. This proportion is within the previously reported range of 0.59%−2.5%, though it falls toward the higher end of that range [4], [5], [6], [7], [8].

**Table 1:** Clinical characteristics of nine cases of subcutaneous emphysema complicating measles.

**Case**	**Age**	**Sex**	**Nutrition**	**Criterion**	**Diagnosis**	**SE onset**	**SE distribution**	**Pneumothorax**	**Management**	**Stay**	**Outcome**	**Transfer**
1	4	M	Severe acute malnutrition	MUAC < 11.5 cm + bilateral pitting edema	SCAP	3	Chest, neck	No	Supportive care	12	Recovered	No
2	6	F	Normal	MUAC > =12.5 cm, no edema	SCAP	4	Chest	No	Supportive care	8	Recovered	No
3	12	M	Moderate acute malnutrition	MUAC 11.5–12.4 cm	SCAP	5	Chest, back	Yes	Needle decompression	15	Recovered after 5 days ICU care; no chest tube	Regional hospital
4	18	F	Severe acute malnutrition	MUAC < 11.5 cm + WHZ < −3	Measles with malaria	4	Chest, neck, face	No	Supportive care	10	Recovered; supplemental O2 for 3 more days	Regional hospital
5	24	M	Normal	MUAC > =12.5 cm	SCAP	2	Chest	No	Supportive care	6	Recovered	No
6	30	F	Severe acute malnutrition	MUAC < 11.5 cm + edema	Measles with malaria	4	Chest, back, neck	No	Supportive care	14	Recovered; no invasive procedures	Regional hospital
7	36	M	Normal	MUAC > =12.5 cm	SCAP	5	Chest	Yes	Needle decompression; chest tube after transfer	18	Recovered; chest tube after transfer	Regional hospital
8	42	F	Moderate acute malnutrition	MUAC 11.5–12.4 cm	SCAP	3	Chest, neck	No	Supportive care	9	Recovered	No
9	48	M	Normal	MUAC > =12.5 cm	Measles with malaria	6	Chest, back	Clinically suspected	Supportive care; transfer for ICU monitoring	11	Died within 48 h after transfer from hypoxemic respiratory failure	Regional hospital

**SCAP =** severe community-acquired pneumonia; **SE** = subcutaneous emphysema; **MUAC =** mid-upper arm circumference; **WHZ** = weight-for-height Z-score; **O2** = oxygen. Maximum humidified nasal oxygen flow ranged from 2 to 6 L/min, approximately 0.5–1.2 L/kg/min, titrated to maintain SpO2 > =92%. Transfer refers to transfer from Bachuma Primary Hospital to a regional hospital for intensive monitoring or higher-level care.

The affected cohort comprised five males and four females with a median age of 24 months. The broader group of 589 hospitalized measles patients ranged from 2 months to 10 years of age, with a median age of 18 months; 496 children (84%) were younger than 5 years. All nine children in this case series were unvaccinated against measles. Regional measles-containing vaccine first-dose coverage in West Omo was estimated at < =45% among children aged 12–23 months, and fewer than 5% of admitted children had documented vaccination. Reported barriers included geographic inaccessibility, vaccine stockouts at health posts, low community awareness and vaccine hesitancy, seasonal migration, and conflict-related displacement. Longitudinal vaccination and refusal data were not available; therefore, we could not determine whether under-vaccination or vaccine hesitancy was increasing over time.

Five children had acute malnutrition. Three met criteria for severe acute malnutrition based on MUAC < 11.5 cm with edema or weight-for-height Z-score < −3 where available, and two met criteria for moderate acute malnutrition based on MUAC 11.5–12.4 cm. MUAC was the primary screening tool; edema and weight-for-height or weight-for-length Z-scores were used when available. Chronic malnutrition or stunting could not be reliably assessed because height-for-age data and longitudinal anthropometric records were unavailable. Eight children had clinical signs of micronutrient deficiency, including oral thrush and dermatosis.

Six children presented with severe community-acquired pneumonia characterized by respiratory distress, tachypnea, and hypoxemia requiring oxygen supplementation. Three had concomitant severe malaria infection. Subcutaneous emphysema manifested a median of 4 days after measles rash onset, with a range of 2–6 days. Only two patients presented with subcutaneous emphysema evident at hospital admission; the remaining seven developed emphysema 1–5 days after hospitalization, typically after at least 10 h of humidified nasal oxygen therapy.

**Fig. 1:**
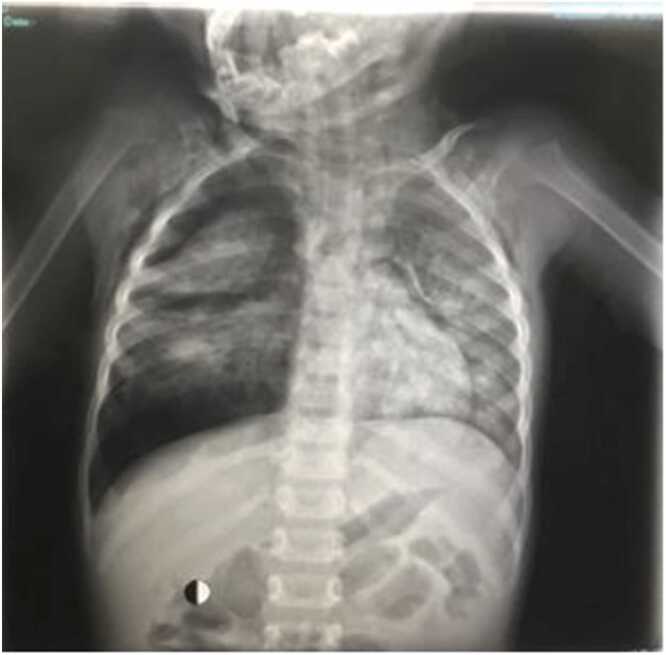
Chest radiograph demonstrating subcutaneous emphysema and pneumomediastinum in a 12-month-old male patient with measles-associated severe community-acquired pneumonia (**Case** 3).

**Fig. 2:**
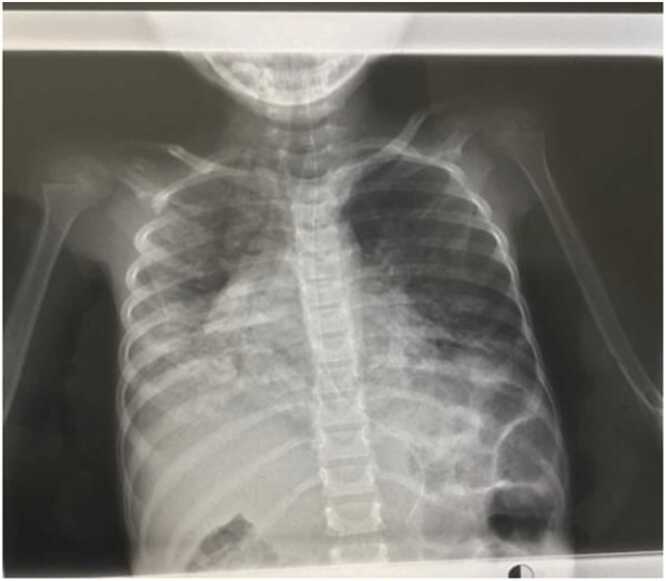
Chest radiograph showing pneumomediastinum with subcutaneous emphysema in a 30-month-old female patient with measles complicated by severe malaria (**Case** 6).

All patients exhibited palpable crepitus on examination. Anterior chest wall involvement was present in all cases, with extension to the back, neck, face, or mandibular regions in several patients. Two patients developed clinical signs consistent with tension pneumothorax requiring emergent needle decompression at Bachuma Primary Hospital. Chest radiography was performed in two patients because of limited imaging availability, confirming subcutaneous emphysema with associated pneumomediastinum (Fig. 1, Fig. 2).

Initial management of severe measles pneumonia consisted of humidified nasal oxygen, vitamin A supplementation, antipyretics, broad-spectrum antibiotics when clinically indicated, and nutritional rehabilitation. Oxygen flow ranged from 2 to 6 L/min, approximately 0.5–1.2 L/kg/min depending on weight and severity, and was titrated to maintain SpO2 > =92%. No patient received CPAP, bag-mask ventilation, or intubation at Bachuma Primary Hospital because advanced respiratory support equipment was unavailable. Following recognition of subcutaneous emphysema, oxygen was continued only as needed for hypoxemia and patients were closely monitored for pneumothorax or clinical deterioration.

Antibiotic coverage was broadened to include anaerobic organisms in five cases because clinicians were concerned about necrotizing pneumonia or lung abscess in severely ill and malnourished children. This practice reflected empirical local clinical judgment in a setting without microbiologic confirmation and should not be interpreted as a standard recommendation for uncomplicated subcutaneous emphysema in the absence of esophageal perforation, necrotizing infection, or lung abscess.

Five patients were transferred from Bachuma Primary Hospital to a regional hospital for intensive monitoring or higher-level care. Cases 3, 4, and 6 recovered without invasive procedures after transfer, although Case 3 required five days of ICU care and Case 4 required supplemental oxygen for three additional days. Case 7 recovered after chest tube placement for pneumothorax at the regional hospital. Case 9, a 48-month-old male, died within 48 h after transfer from hypoxemic respiratory failure with severe measles pneumonia and clinically suspected pneumothorax. Overall, eight patients recovered and one died.

## Discussion

This case series documents subcutaneous emphysema in nine children during a measles outbreak in rural Ethiopia. The observed incidence of 1.52% is at the higher end of previously reported outbreak ranges, rather than clearly exceeding them. The series is clinically important because it occurred in a setting marked by young age, low vaccination coverage, malnutrition, severe pneumonia, limited imaging, and limited respiratory support.

The clinical pattern is consistent with air-leak physiology related to the Macklin phenomenon, in which alveolar rupture allows air to dissect along bronchovascular sheaths into the mediastinum and subcutaneous tissues [9], [10]. In measles, viral pneumonitis, secondary infection, severe cough, and respiratory distress may increase alveolar pressure and weaken tissue integrity, particularly in children with malnutrition.

Malnutrition was common in this series and may have increased vulnerability to severe measles pneumonia and air-leak complications. Three children had severe acute malnutrition and two had moderate acute malnutrition. We revised the malnutrition definition to avoid BMI-for-age Z-scores in children younger than 5 years and instead used MUAC, edema, and weight-for-height or weight-for-length Z-scores when available. This is particularly relevant in outbreak settings where MUAC is often the most feasible screening tool.

Severe respiratory disease was common, with six children presenting with severe community-acquired pneumonia. Among children hospitalized with complicated measles, this finding is not unexpected, but it remains clinically meaningful because pneumonia contributes substantially to measles mortality and creates the cough and respiratory effort that can precipitate air-leak phenomena [12], [13], [17].

Humidified nasal oxygen therapy may have contributed in some cases, but it should be interpreted as a possible cofactor rather than a proven cause. Seven children developed subcutaneous emphysema after at least 10 h of oxygen therapy, with maximum flow rates of 2–6 L/min, estimated at 0.5–1.2 L/kg/min. Oxygen was clinically necessary for hypoxemia, and the primary practical implication is careful titration to the lowest flow that maintains adequate saturation, paired with repeated examination for crepitus and signs of pneumothorax.

The age distribution of the cases reflected the broader hospitalized measles population. The median age among the nine children was 24 months, compared with 18 months among all 589 hospitalized patients, 84% of whom were younger than 5 years. This supports the interpretation that young children were the primary affected population in the outbreak rather than a uniquely older or younger subgroup within the air-leak cases.

Transfer outcomes add important context. Four of the five transferred children recovered, including one who required chest tube placement after transfer. The only death occurred after transfer in a child with severe measles pneumonia, clinically suspected pneumothorax, late presentation, and limited access to ICU-level respiratory support. Because no patient was lost to follow-up, the reported mortality in this series reflects known outcomes for all nine children.

The transfer outcomes should be interpreted in light of the practical risks of moving critically ill patients in resource-limited settings [18]. The fatal case in this series is consistent with reports that severe complications, delayed care, young age, and undernutrition contribute to measles mortality in outbreak settings [19].

**Clinical Implications:** Clinicians managing measles patients in resource-limited settings should maintain vigilance for subcutaneous emphysema, particularly in young children with malnutrition, severe pneumonia, and oxygen requirement. Regular physical examination for crepitus is essential when radiography is unavailable. Facilities managing measles outbreaks should have protocols for oxygen titration, recognition of pneumothorax, emergency needle decompression, nutritional rehabilitation, and timely referral.

**Limitations**: This study is limited by its small sample size, retrospective design, limited radiographic confirmation, limited microbiologic testing, and the constraints of a primary hospital without advanced respiratory support. Oxygen flow estimates in L/kg/min were approximate because weights were not uniformly recorded contemporaneously with peak oxygen use. Chronic malnutrition or stunting could not be reliably assessed because height-for-age and longitudinal anthropometric records were unavailable. Longitudinal vaccination and refusal data were also unavailable, so trends in vaccine access or hesitancy over time could not be assessed. The findings should therefore be interpreted as descriptive and hypothesis-generating rather than causal.

## Conclusion

Subcutaneous emphysema complicated measles in 1.52% of hospitalized children during this rural Ethiopian outbreak, a rate at the higher end of previously reported ranges. The cases occurred in a vulnerable population marked by low vaccination coverage, young age, malnutrition, severe pneumonia, and limited access to imaging and advanced respiratory support. Early recognition of crepitus, careful oxygen titration, readiness for emergency decompression, nutritional rehabilitation, and clear transfer pathways are important in outbreak settings. Prevention through measles vaccination, improved vaccine access, and attention to childhood malnutrition remains central to reducing avoidable morbidity and mortality.

## CRediT authorship contribution statement

**Mekbul Mohammed Aman:** Supervision. **Leul M. Manyazewal:** Writing – review & editing, Validation, Software. **Teferi Mikiyas Gifawosen:** Writing – original draft, Formal analysis. **Abel A. Gelan:** Resources. **Caleb M. Hailemariam:** Writing – review & editing. **Nahom D. Gerer:** Writing – review & editing, Investigation, Conceptualization.

## Consent

Written informed consent was obtained from the legal guardians of all nine pediatric patients included in this case series for the publication of their clinical details, including demographic information, clinical presentations, management approaches, and outcomes.

## Ethics approval

Ethics approval was waived by the institutional review body as this analysis was part of outbreak response.

## Funding

None received.

## Originality & ethical compliance

This manuscript is original research that has not been previously published or submitted to any other journal. The case series complies with international ethical standards for medical research and publication. Written informed consent was obtained from the legal guardians of all patients for publication of their clinical information. Patient confidentiality has been maintained throughout data collection, analysis, and manuscript preparation, with no identifying information included in the published material.

## Declaration of Competing Interest

No financial, institutional, or personal conflicts of interest influenced the preparation, conduct, or submission of this work. The authors have no financial relationships with any organizations that might have an interest in the submitted work, and no other relationships or activities that could appear to have influenced the submitted work.
